# Evolution of plastid genomes of *Holcoglossum* (Orchidaceae) with recent radiation

**DOI:** 10.1186/s12862-019-1384-5

**Published:** 2019-02-26

**Authors:** Zhang-Hai Li, Xiao Ma, De-Yi Wang, Yun-Xia Li, Cheng-Wang Wang, Xiao-Hua Jin

**Affiliations:** 1 State Key Laboratory of Systematic and Evolutionary Botany, Institute of Botany, Chinese Academy of Sciences, Beijing, China; 2 Southeast Asia Biodiversity Research Institute, Chinese Academy of Science (CAS-SEABRI), Nay Pyi Taw, Myanmar; 3 University of Chinese Academy of Sciences, Beijing, China; 4 Fujian Agriculture and Forest University, Shanxiadian Road 15, Changshan District, Fuzhou, 350002 Fujian China; 5 Nanchang University, Xuefu Road 999, Honggutang District, Nanchang, Jiangxi China

**Keywords:** *Holcoglossum*, Plastid genome, NDH complex, Divergence hotspot, Intraspecific variation, Tandem repeat, Hairpin inversion

## Abstract

**Background:**

The plastid is a semiautonomous organelle with its own genome. Plastid genomes have been widely used as models for studying phylogeny, speciation and adaptive evolution. However, most studies focus on comparisons of plastid genome evolution at high taxonomic levels, and comparative studies of the process of plastome evolution at the infrageneric or intraspecific level remain elusive. *Holcoglossum* is a small genus of Orchidaceae, consisting of approximately 20 species of recent radiation**.** This made it an ideal group to explore the plastome mutation mode at the infrageneric or intraspecific level.

**Results:**

In this paper, we reported 15 complete plastid genomes from 12 species of *Holcoglossum* and 1 species of *Vanda.* The plastid genomes of *Holcoglossum* have a total length range between 145 kb and 148 kb, encoding a set of 102 genes. The whole set of *ndh*-gene families in *Holcoglossum* have been truncated or pseudogenized. Hairpin inversion in the coding region of the plastid gene *ycf2* has been found.

**Conclusions:**

Using a comprehensive comparative plastome analysis, we found that all the indels between different individuals of the same species resulted from the copy number variation of the short repeat sequence, which may be caused by replication slippage. Annotation of tandem repeats shows that the variation introduced by tandem repeats is widespread in plastid genomes. The hairpin inversion found in the plastid gene *ycf2* occurred randomly in the Orchidaceae.

**Electronic supplementary material:**

The online version of this article (10.1186/s12862-019-1384-5) contains supplementary material, which is available to authorized users.

## Background

The plastid is a semiautonomous organelle that evolved from cyanobacteria by endosymbiosis [1]. During the course of evolution, the coding capacity of plastid genomes (plastomes) has experienced drastic reductive evolution with gene loss or transfer to the nucleus [2–4]. The genes reserved in plastomes are usually necessary for the chloroplast to perform its normal functions, including approximately 80 unique protein-coding genes, 30 tRNA genes and 4 rRNA genes. In addition to highly conserved gene content, the organization of the plastome in higher plants is remarkably conserved, which is characterized by two large inverted repeat regions (IRA and IRB) separated by two single copy regions with different lengths, known as a large single copy region (LSC) and a small single copy region (SSC) [3, 5–7].

Benefiting from the advances in next-generation sequencing, more plastid genomes have been sequenced, and there are more than 2800 records of eukaryotic plastid genomes available in the NCBI database (https://www.ncbi.nlm.nih.gov/genomes/GenomesGroup.cgi?opt=plastid&taxid=2759 last accessed May 30, 2018). Due to their frequent sequencing and wide availability, plastid genomes have been used as models in genetic variation studies, encompassing both micro- and macro-evolutionary events across all lineages of plants [8–14]. However, previous studies have mostly focused on comparisons of plastid genome evolution at higher taxonomic levels (e.g., across genera or families or orders) or between autotrophic and heterotrophic plants, which may have phylogenetic sampling ‘gaps’ or evolutionary route ‘gaps’ [15]. This may cause key steps in the process of plastome evolution at the infrageneric or intraspecific level to remain elusive.

The genus *Holcoglossum* Schltr. (Vandeae, Orchidaceae) consists of approximately 20 species that are mainly distributed in southwestern China and neighbouring regions [16–24]. *Holcoglossum* has two diversity centres, one in the tropical region and the other in the temperate alpine region of the Hengduan Mountains (HDM), with an elevation of over 2000 m [20, 23, 25]. At least six species of *Holcoglossum* are distributed in the HDM, five of which are restricted to this area [23]. Biogeographic analyses and molecular phylogeny suggest that *Holcoglossum* dispersed from tropical regions to the HDM and then radiated there [23]. Previous results indicated that the pendent growing pattern [23] and laterocytic and polarcytic stomata are perhaps ecological adaptations to the strong winds and ample rains in the alpine region of the HDM [26]. Rapid changes in temperature and weather conditions are major challenges for the species living in temperate alpine regions in the HDM. Previous results indicated that plastid genes of *Cardamine resedifolia* (Brassicaceae) experienced more intense positive selection than those of the low altitude *C. impatiens*, possibly as a consequence of adaptation to high altitude environments [12].

Here, using comparative plastid genomes of 15 complete plastome sequences of 12 species of *Holcoglossum* and 1 species of *Vanda*, we aim to (1) understand the evolution of the plastid genome in *Holcoglossum* and (2) investigate the evolutionary pattern of the plastid genome at infrageneric and intraspecific levels.

## Methods

### Taxa sampling, DNA isolation, library preparation, and sequencing

In this study, we sampled and sequenced 12 species of *Holcoglossum,* including 2 individuals of *H. flavescens* and *H. nujiangense*, and 1 species of *Vanda*. Two plastomes of *Neofinetia* were downloaded from NCBI (Table 1) as outgroups. Fresh leaves, stems and flowers were collected in the field and preserved in silica gel as well as frozen at − 20 °C. Total DNA was isolated using a modified cetyltrimethyl ammonium bromide (CTAB) protocol [27]. DNA with concentrations greater than 100 ng/ml was sheared to 500 bp using Covaris M220. Sequencing libraries were prepared using the NEBNext Ultra DNA Library Prep Kit (according to the manufacturer’s protocol) for subsequent paired-end sequencing on an Illumina HiSeq 2500 at the Institute of Botany, Chinese Academy of Sciences.

**Table 1:** Basic information of plastid genomes used in this study

Species	Mean coverage	Length (bp)				GC Content (%)	No. vouchers specimen or NCBI accession
		Total	LSC	SSC	IR		
*Holcoglossum nujiangense*_S1_S16	220	146,487	82,955	11,916	25,808	35.4	Jin Xiaohua 6930
*Holcoglossum nujiangense*_S5_S9	539	146,395	82,873	11,906	25,808	35.4	Jin Xiaohua 10,897
*Holcoglossum weixiense*	205	146,597	82,981	12,000	25,808	35.4	HK Kadoorie Program Team 3490
*Holcoglossum sinicum*	507	145,909	82,658	11,635	25,808	35.4	Jin Xiaohua 14,683
*Holcoglossum flavescens* _S2_S18	183	146,863	83,288	11,959	25,808	35.3	Jin Xiaohua 8943
*Holcoglossum flavescens*_S5_S10	489	146,763	83,188	11,959	25,808	35.4	Jin Xiaohua 15,165
*Holcoglossum rupestre*	75	147,163	83,575	11,936	25,826	35.3	Jin Xiaohua 9015
*Holcoglossum quasipinifolium*	131	147,063	83,440	12,079	25,772	35.4	JXH028
*Holcoglossum lingulatum*	151	146,525	83,713	11,274	25,769	35.5	Jin Xiaohua 9491
*Holcoglossum nagalandensis*	357	146,826	83,763	11,477	25,793	35.3	Jin Xiaohua 10,522
*Holcoglossum amesianum*	425	148,074	84,250	12,026	25,899	35.3	Jin Xiaohua 9419
*Holcoglossum himalaicum*	752	145,207	83,712	11,413	25,041	35.3	Jin Xiaohua 9496
*Holcoglossum wangii*	209	147,170	83,846	11,594	25,866	35.4	Jin Xiaohua 13,881
*Holcoglossum subulifolium*	360	146,930	83,398	11,802	25,865	35.5	Jin Xiaohua 13,614
*Neofinetia falcata*	_	146,497	83,809	11,775	25,456	35.3	NC_036372
*Neofinetia richardsiana*	_	146,498	83,809	11,775	25,457	35.3	NC_036373
*Vanda brunnea*	191	149,216	85,783	11,713	25,860	35.3	Jin Xiaohua 13,059

### Plastome assembly and annotation

Plastome assembly and annotation followed the methods of Feng et al. (2016) [10]. In short, raw reads were trimmed and filtered with NGSQCTOOLKIT v 2.3.3 [28], and bases with a PHRED quality lower than 20 were trimmed. All trimmed reads shorter than 70 bp were discarded. The filtered reads were mapped to the plastome of *Calanthe triplicata* (https://www.ncbi.nlm.nih.gov/nuccore/NC_024544.1) in Geneious v10.2.2 (http://www.geneious.com, last accessed June 4, 2017) to filter reads matching the reference genomes. De novo assemblies were constructed in VELVET [29] with several K-mer values, and contigs from each assembly were merged in Geneious and combined into scaffolds using the default parameters (minimum overlap 20 bp, minimum similarity 70%). Alternatively, contigs from both assemblies (Geneious or Velvet) were merged in SSPACE [30] to form scaffolds/draft genomes. IR boundaries for each draft plastome were confirmed by BLAST [31], with the first and last sequences (approximately 50 bp) of the draft plastome used as search terms. The finished plastomes were annotated by using DOGMA with an e value of 5% and identity thresholds of 60 and 80% for protein-coding genes and tRNAs, respectively [32]. Smaller exons (< 30 bp) were manually annotated by local BLAST in Geneious. The initiation codon, termination codon, and other annotation errors for each gene were revised in Sequin and exported as GenBank files.

### DNA alignment and phylogenetic analysis

We generated multiple sequence alignments of whole plastid genomes using MAFFT under the automatic model selection option with some manual adjustments [33]. At the same time, 68 protein-coding sequences were exported from plastomes in Geneious. The protein-coding sequences were aligned at the codon level with the option “-codon” using MUSCLE [34] in MEGA v7.0.2 [35]. Stop codons were removed from the sequences prior to alignment. The phylogenetic trees were reconstructed based on the nucleotide data of whole plastid genomes with the GTRGAMMA model using RAxML v8.0.9 [36] in the CIPRES Science Gateway [37], and branch support was assessed using 1000 standard bootstrap replicates.

### Sequence divergence analysis

We compared the overall similarities among different plastomes in *Holcoglossum* using *H. subulifolium* with one IR region removed as a reference. The sequence identity of the *Holcoglossum* plastid genomes was plotted using the mVISTA program with the LAGAN mode [38]. To screen variable characters within *Holcoglossum*, the average number of nucleotide differences (K) and total number of mutations (Eta) were determined to analyse nucleotide diversity (Pi) using DnaSP v6.10.04 [39]. The step size was set to 200 bp, with a 500 bp window length.

The complete plastomes of two *H. flavescens* individuals and two *H. nujiangense* individuals were aligned in Geneious with the MAFFT algorithm, and differences were identified by using the “Find Variations/SNPs” function and checked individually. We recorded substitutions and indels separately, as well as their location in the chloroplast genome.

Since all of the indels in intraspecific variation are caused by the copy number variation of the short repeat sequence, as shown in our results, we further explored whether the tandem repeat also contributed to interspecific plastid genome variation. We located and annotated the tandem repeats on the multiple sequence alignment matrix of *Holcoglossum* plastome with Phobos [40] in Geneious.

### Molecular evolutionary pattern analysis of plastid genes

To explore the selection patterns and identify positive selection on the protein-coding genes, we use two models, model M0 and a branch-site model, implemented in the PAML Codeml program [41]. The codon frequencies were determined by the F3 × 4 model. Twenty-eight genes with too few variable sites were not examined (Additional file 1: Table S2). Alignment gaps and uncertainties were deleted to avoid false positives [42].

The model M0 (model = 0, Nsites = 0, which assumes no site-wise or branch-wise dN/dS variation) estimates the rates of synonymous (dS) and non-synonymous substitutions (dN) and the dN/dS value of each gene, which can be an indication of the selection pattern.

The branch-site model (model = 2, Nsites = 2, fixed omega = 0, omega = 2) was used to detect evidence of positive selection on specific sites along a specific lineage. The goal of our study was to explore the role of positive selection in the adaptive patterns of *Holcoglossum* adapted to tropical regions and temperate alpine regions; thus, the tropical clade and alpine clade were used to perform the selection analyses. The likelihood ratio test (LRT) with a χ^2^ distribution was used to determine which models were significantly different from the null model (model = 2, Nsites = 2, fixed omega = 1, omega = 1) at a threshold of *P* < 0.05. The Bayes empirical Bayes (BEB) method was used to statistically identify sites under positive selection with posterior probabilities ≥0.95 [43].

## Results

### Plastome structure and phylogenomics of *Holcoglossum*

**Fig. 1:**
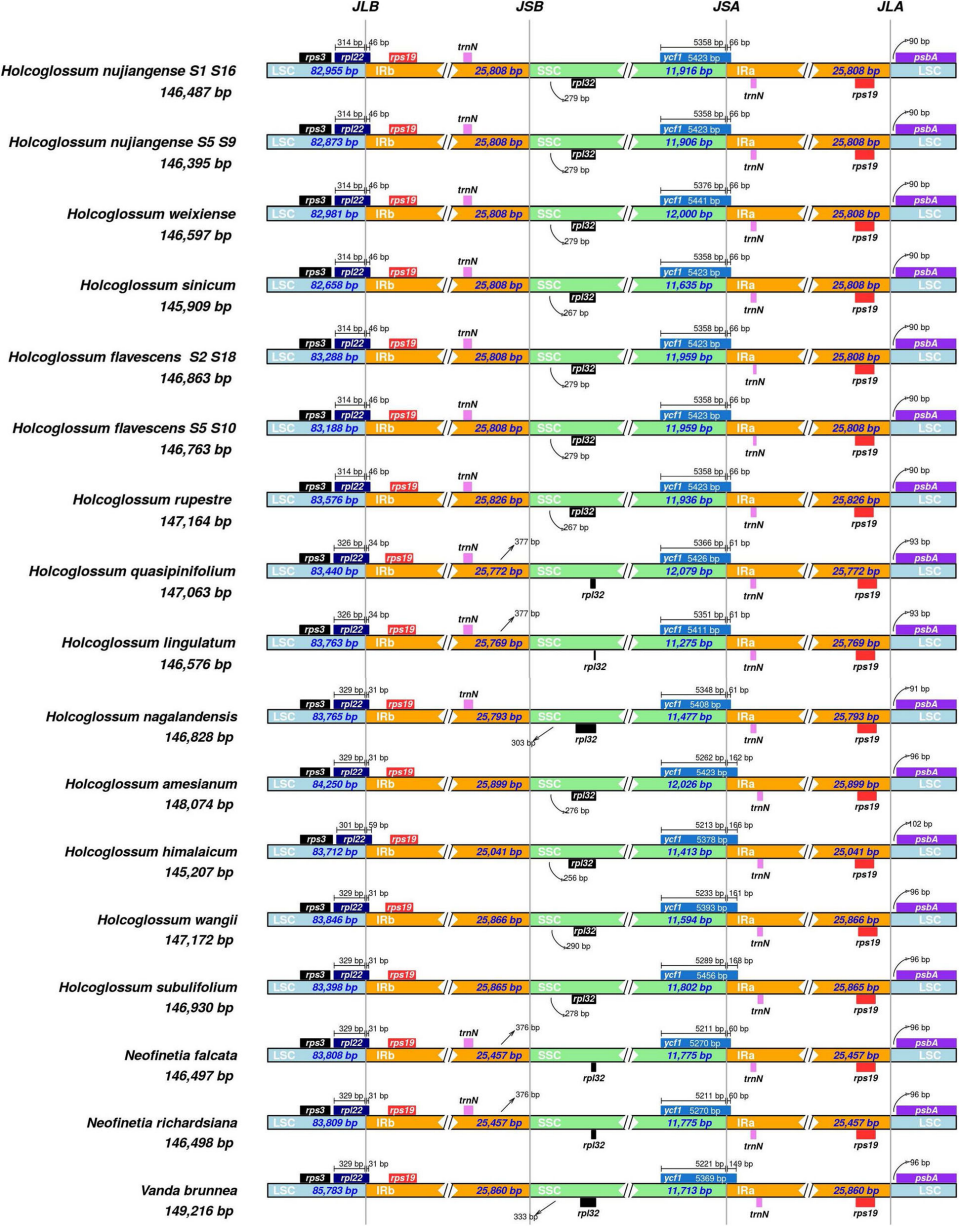
Comparison of the LSC, IR, and SSC junction positions in *Holcoglossum* and three outgroup plastid genomes

In the present study, 14 complete plastomes of 12 species of *Holcoglossum* and 1 species of *Vanda* were obtained for the first time. These plastomes showed the typical quadripartite structure of most angiosperms. The plastomes of *Holcoglossum* had a total length range between 145,207 bp in *H. himalaicum* and 148,074 bp in *H. amesianum*. The length variation of the *Holcoglossum* plastomes observed here was low (145–148 kb). The expansion and contraction of the inverted repeat regions usually contribute to variation in the length of plastomes. In this study, we found that the IR/SSC boundary was located differently among the 12 *Holcoglossum* species, but the location of the boundary and length of the IR regions only showed moderate variation (Table 1), and there was no obvious phylogenetic implication of extension/contraction of IRs among the *Holcoglossum* plastomes (Fig. 1).

All of the sequenced *Holcoglossum* plastomes are highly conserved in structure compared to most angiosperms, sharing the common typical quadripartite structure comprising two copies of IR (25,041–25,899 bp) separated by the LSC (82,658–84,250 bp) and SSC (11,275–12,079 bp) regions (Table 1). The overall GC content was between 35.3–35.5% (Table 1), which is similar to the other Orchidaceae plastomes sequenced thus far [44, 45]. The *Holcoglossum* plastomes encoded an identical set of 102 genes, of which 85 were unique and 17 were duplicated in the IR regions. The 102 genes contained 68 protein-coding genes, 30 tRNA genes, and 4 rRNA genes (Additional file 2: Table S1). Functional cp-*ndh* genes have been lost or pseudogenized in all *Holcoglossum* species.

Phylogenetic analyses indicated that *Holcoglossum* is monophyletic and subdivided into three strongly supported clades (ML bootstrap =100%): the tropical clade (TC) with five species, the alpine clade (AC) with five species and the HC clade with two species (Additional file 3: Figure S1). All of the nodes among the lineages in our tree were strongly supported by ML bootstrap values ≥94% (Additional file 3: Figure S1). Our results indicated that *H. amesianum* and *H. naglandensis* are sister groups forming a sister clade to *H. himalaicum* and *H. wangii*.

### Intraspecific plastome variation and mutation hotspots of *Holcoglossum* plastomes

Comparing plastomes of two individuals of *H. flavescens*, we found 17 SNPs, 1 single nucleotide indel and 3 multi-nucleotide indels ranging from 14 to 57 bp in *H. flavescens*. Between the two individuals of *H. nujiangense*, 8 SNPs, 3 single nucleotide indels and 5 multi-nucleotide indels of 3–36 bp length have been found (Table 2). All of the SNPs and indels are located in the LSC and SSC regions, and all of the indels contributing to intraspecific variation are caused by the copy number variation of short repeat sequences.

**Table 2:** Intraspecific variation between two individuals of *H. flavenscens* and *H. nujiangense*

*Holcoglossum flavenscens*				*Holcoglossum nujiangense*			
Position	Varitation type	Location	Location type	Position	Varitation type	Location	Location type
895	G/A	*psbA*	coding	4271	Indel(3 bp)	*trnK*	IGS
9882	A/G	*trnG*	IGS*	8011	./T	*psbK_psbI*	IGS
10,370	T/C	*trnR_atpA*	IGS	20,023	A/G	*rpoC2*	coding
17,761	T/G	*rpoC2*	coding	21,021	A/C	*rpoC1*	coding
33,914	C/A	*trnT_psbD*	IGS	21,031	G/A	*rpoC1*	coding
36,702	A/C	*trnS_psbZ*	IGS	28,263	Indel(20 bp)	*rpoB_trnC*	IGS
43,116	C/T	*psaA_ycf3*	IGS	32,903	./A	*trnE_trnT*	IGS
49,010	C/A	*trnL_trnF*	IGS	33,559	A/G	*trnT_psbD*	IGS
49,020	Indel(28 bp)	*trnL_trnF*	IGS	33,845	Indel(19 bp)	*trnT_psbD*	IGS
49,378	Indel(57 bp)	*trnF_trnV*	IGS	49,670	Indel(36 bp)	*trnF_trnV*	IGS
49,514	Indel(14 bp)	*trnF_trnV*	IGS	56,132	G/T	*rbcL_accD*	IGS
49,981	C/A	*trnV*	IGS	79,512	T/C	*rps8_rpl14*	IGS
56,483	A/G	*accD*	coding	81,511	./T	*rpl14_rps3*	IGS
64,283	A/C	*psbE_petL*	IGS	111,832	Indel(10 bp)	*ccsA_psaC*	IGS
67,306	T/C	*rpl20*	coding	112,042	T/C	*ccsA_psaC*	IGS
69,392	T/.	*clpP*	IGS	119,788	G/A	*ycf1*	coding
73,253	G/A	*psbB/psbT*	IGS				
73,948	A/G	*psbH/petB*	IGS				
110,107	G/T	*rpl32_trnL*	IGS				
112,694	A/C	*ccsA_psaC*	IGS				
120,678	T/C	*ycf1*	coding				

*IGS: Inter-Genic Sequence

**Fig. 2:**
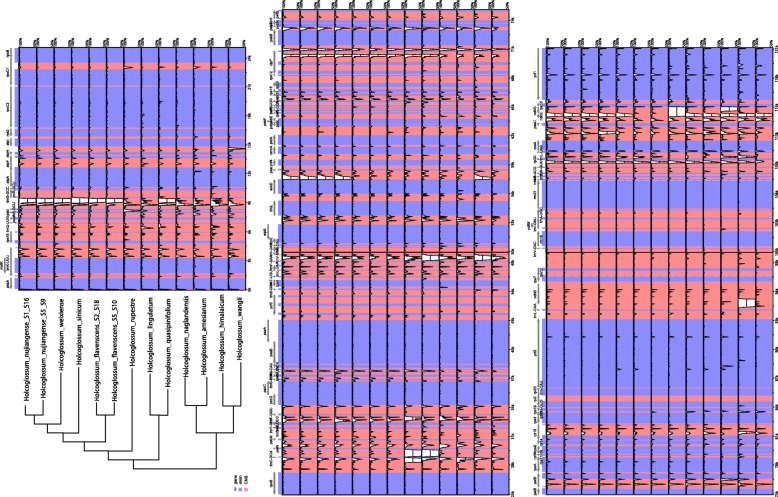
mVISTA percent identity plot of available *Holcoglossum* plastomes using *H. subulifolium* as a reference. The vertical scale indicates the percentage of identity ranging from 50 to 100%. Coding regions are in blue, and noncoding regions are in red. Cladogram redrawn from Additional file 3: Figure S1, branch lengths are not representative of evolutionary changes

**Fig. 3:**
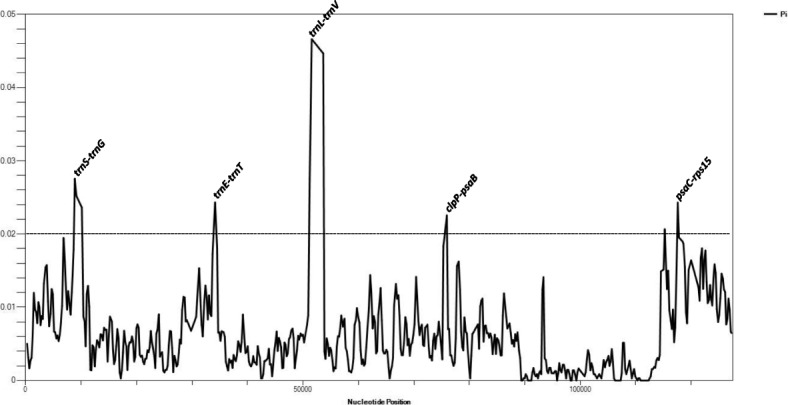
Sliding window analysis of the whole plastid genomes of *Holcoglossum* taxa. The 5 mutation hotspot regions (Pi > 0.02) are annotated

The border regions of LSC/IRB, IRB/SSC, SSC/IRA, and IRA/LSC are usually highly variable even between closely related species [46, 47]. Therefore, we compared and visualized the exact IR border positions and their adjacent genes among the *Holcoglossum* chloroplast genomes and the reference genome using the IRscope online program [48]. The results showed that the genes *trnN-rpl32-ycf1* and *rpl22-rps19-psbA* were located in the junctions of the SSC/IR and LSC/IR regions. The *ycf1* gene spans the SSC/IRA region and extends to the IR region from 61 to 168 bp (Fig. 1). The mVISTA percent identity plot and slide window analysis show that the most divergent regions are located in the *trnS-trnG*, *trnE-trnT*, *trnL-trnV*, *clpP-psbB* and *psaC-rps15* regions in the *Holcoglossum* plastome (Figs. 2 and 3).

### Molecular evolutionary pattern of *Holcoglossum* plastid genes

Most of the plastid genes in *Holcoglossum* are under strongly negative selection with a very low ω value (ω < 0.5), yet the genes *ycf2* and *ycf1* of uncertain function are under neutral selection with a ω value near to 1.0; the only gene found under positive selection is *psbK* with a high ω value (ω = 1.92088) (Additional file 1: Table S2). The branch-site model analysis does not detect any site under positive selection when the alpine clade is set as the foreground branch, while there are 14 sites in *ycf2* and 2 sites in the *ycf1* gene have been detected theoretically under positive selection (as the Bayes Empirical Bayes probability > 0.95) when the tropical clade is set as the foreground branch (Additional file 4: Table S3).

## Discussion

### Phylogeny of Holcoglossum

The phylogenetic relationships among the major lineages of *Holcoglossum* based on plastomes were essentially in agreement with the results of Xiang et al. [24] based on four markers (*matK*, *trnH-psbA*, *trnL-F*, and nuclear ITS sequences) with the exception of the placement of *H. amesianum*. Our results indicated that *H. amesianum* and *H. naglandensis* are sister groups forming a sister clade with *H. himalaicum* and *H. wangii.* However, *H. amesianum* had been placed in a sister clade to the clade formed by *H. naglandensis*, *H. himalaicum* and *H. wangii* but with low support (PP = 0.78, BS < 50) in previous results [24]. The difference may be due to the different taxonomic sampling in the two studies or the markers used in the previous study being unable to resolve the phylogenetic relationships in *Holcoglossum*.

### Hairpin inversion in plastid gene *ycf2*

The plastid gene *ycf2* is a large yet functionally undefined ORF in land plants. Nucleotide sequence similarity among land plant *ycf2* is extraordinarily low compared to other plastid-encoded genes, being less than 50% across bryophytes, ferns, and seed plants [5]. When we aligned the protein coding gene *ycf2* of *Holcoglossum*, we found a short inversion mediated by a 17 bp inverted repeat sequence located down- and up-stream in *H. flavescens*, *H*. *quasipinifolium*, *H*. *amesianum* and *H*. *naglandensis* (Additional file 5: Figure S2). To understand whether this inversion occurred randomly, we analysed it across the Orchidaceae family. We found that this motif is conserved at the sequence level in Orchidaceae but is inversely randomly mediated by the hairpin structure. In some species, this motif has been lost or disrupted (Additional file 6: Figure S3).

Previous studies show that most stem-loop structures involving small inversions occur in close proximity to the stop codons of genes and have the function of stabilizing the corresponding mRNA molecules [49], and the majority of the small inversions were located downstream of adjacent genes with a tail-to-tail orientation [50]. However, the hairpin inversion in the plastid gene *ycf2* found in this study is located in the coding region, occurring randomly and being disrupted in some species. These results indicated that this motif may not be pivotal for *ycf2* to exercise its function, and this needs to be revised with a broader sample.

### Intraspecific variation of plastomes

Most of the SNPs found between the two different individuals of *Holcoglossum* are located in intergenic regions. We found 5 SNPs located in the coding region of *psbA*, *rpoC2*, *accD*, *rpl20* and *ycf1* in *H. flavescens*, among which the SNPs located in *rpoC2* and *accD* lead to a nonsynonymous mutation between these two individuals. In *H. nujiangense*, we found 1 synonymous mutation SNP in *rpoC2*, 2 nonsynonymous mutation SNPs in *rpoC1*, and 1 nonsynonymous mutation SNP in *ycf1*. Interestingly, all of these intraspecific variation sites in coding regions are usually conserved between species. All 3 indels found in *H. flavescens* are located in the intergenic region (1 in *trnL-trnF*, 2 in *trnF-trnV*); the 5 indels found in *H. nujiangense* are located in the intron region of *trnK*, the intergenic region of *rpoB-trnC*, *trnT-psbD*, *trnF-trnV* and *ccsA-psaC*. Comparative analysis found that all indels are caused by the copy number variation of the short repeat sequence, which may be caused by replication slippage (Additional file 7: Figure S4). This is in line with a previous study that found that the intraspecific variation in the chloroplast genome of *Astragalus membranaceus* was due to an extra copy of the “TATATATTTA” repeat [51], and the vast majority of mutations in the spontaneous plastome mutants of *Oenothera* are indels originating from DNA replication slippage events [52]. Furthermore, the location of intraspecific variation loci shows that most variations in these two species are species-specific except for the variation in the mutation hotspot region *trnL-trnV*. These intraspecific loci represent potential markers that can be used to distinguish closely related varieties of specific taxa. However, further population genetic studies are still needed to determine whether intraspecific genetic diversity is linked to geographic ranges or the intrinsic characteristics of the taxonomic group.

### Tandem repeat sequences contribute to plastid genome evolution

DNA tandem repeats (TRs) are not just popular molecular markers but are also important genomic elements from an evolutionary and functional perspective [53–56]. Because all the indels found in intraspecific variation are caused by the copy number variation of the short repeat sequence, as shown in our results, we further explored whether the tandem repeat also contributed to interspecific plastid genome variation. We located and annotated the tandem repeats on the multiple sequence alignment matrix of the *Holcoglossum* plastome with Phobos [40] in Geneious. Our results indicated that the mutation hotspot regions are always accompanied by densely distributed tandem repeats (Additional file 8: Figure S5), which indicates that the tandem repeat sequences play an important role in plastid genome variation between closely related species. This finding is consistent with the observation that nearly all detected mutations in the spontaneous plastome mutants of *Oenothera* could be associated with repetitive elements [52].

Furthermore, we found that in the plastid gene *ycf2*, a 15 bp extra copy of “TCGATATTGATGATA” is synapomorphic for the TC clade, whereas the possession of the 9 bp duplication of “ATGATAGTA” is synapomorphic for the HC plus AC clade, with a reversal (secondary loss) in *H. lingulatum* (Additional file 9: Figure S6). Therefore, the HC clade can be referred to as the “intermediate clade” as suggested by Xiang et al. [24]. However, whether these repeat regions have contributed to the adaption to different habitats (here referring to tropical and temperate alpine regions) remains to be verified.

### Positive selection on photosynthetic chloroplast genes

Understanding the patterns of divergence and adaptation among the members of a specific phylogenetic clade can offer important clues about the forces driving its evolution [12, 57–59]. In this study, we detected some positive selective signals in the tropical clade, but sites under positive selection are quite rare and mainly detected in the *ycf1* and *ycf2* genes. This may be because adaptive modifications to other abiotic stresses targeting genes in the nucleus were sufficient to maintain homeostasis for photosynthesis since there are a variety of strategies for plants to adapt to the environment, so there is no need for adaptive evolution of chloroplast-encoded genes [60, 61].

### NDH complex coding genes lost in *Holcoglossum* plastome

The chloroplast NAD(P)H-dehydrogenase-like (NDH) complex is located in the thylakoid membrane and plays an important role in mediating photosystem I cyclic electron transport (PSI-CET) and facilitating chlororespiration [62, 63]. Loss of the cp-*ndh* genes is widely reported in heterotrophic species because they do not need to synthesize organic carbon through photosynthesis by themselves [10, 11, 13, 64, 65]. However, as more plastid genomes have been sequenced, some autotrophic plants, such as some species of Pinales, Geraniaceae and Orchidaceae, have also been reported to lose almost the entire set of cp-*ndh* genes [66–70]. In our study, we also found that all of the cp-*ndh* genes were truncated or pseudogenized in the *Holcoglossum* plastid genome.

The loss of plastome genes may be due to transfer to the nucleus, substitution of a nuclear encoded mitochondrial targeted gene or substitution of a nuclear gene for a plastid gene. Translocation of *ndh* genes to the chondriome in *Cymbidium* has been reported, and different levels of *ndh* gene degradation among even closely related species in *Cymbidium* may be due to multiple bidirectional intracellular gene transfers between two organellar genomes [71]. As there is an alternative PSI cyclic electron transport pathway: the proton gradient regulation 5 (PGR5)/PGR5-like photosynthetic phenotype 1 (PGRL1)-dependent antimycin A-sensitive pathway [72–74], especially under high light conditions, the NDH1 pathway would be minor, while the PGR5 pathway would be dominant [63, 75]. The NDH complex may not be necessary for some plants. Using comparative genome analyses, Lin et al. found that nuclear NDH-related genes are also lost in orchids without cp-*ndh* genes [76].

## Conclusions

In this study, we reported 15 completed plastid genomes using Illumina sequencing technology via a reference-guided assembly. These plastid genomes were highly conserved, and the whole set of *ndh*-gene families was truncated or pseudogenized. The five mutation hotspot regions were identified across the *Holcoglossum* plastid genomes, which could serve as potential markers for phylogenetic and population genetic studies. We further investigated the intraspecific variation of indels and substitutions in two species, and potentially diagnostic variations have been found in the plastomes of different individuals. A hairpin inversion in the coding region of the plastid gene *ycf2*, which occurred randomly in Orchidaceae, was found in this study. We additionally found evidence that the tandem repeat sequences contribute to the evolution of the plastid genome not only in the intergenic region but also in the coding region.

## Additional files

Additional file 1: **Table S2.** Statistic of substitution sites and ω values of *Holcoglossum* plastid genes. (XLSX 15 kb)

Additional file 2: **Table S1.** List of genes identified in the plastid genomes of *Holcoglossum*. (DOCX 26 kb)

Additional file 3: **Figure S1.** Maximum Likelihood phylogenetic tree of *Holcoglossum* based on the whole plastid genome except for one invert repeat region. Bootstrap support is indicated on the nodes. (PDF 171 kb)

Additional file 4: **Table S3.** Detected positive selection sites in the plastid genes of TC clade *Holcoglossum* species. (DOCX 19 kb)

Additional file 5: **Figure S2.** Hairpin inversion of *ycf2* in *Holcoglossum*. (PNG 236 kb)

Additional file 6: **Figure S3.** Hairpin inversion of *ycf2* in Orchidaceae. (PDF 316 kb)

Additional file 7: **Figure S4.** Intraspecific variation resulting from tandem repeats in *H. flavenscens*. (PNG 201 kb)

Additional file 8: **Figure S5.** Tandem repeat annotated to the whole plastid genome (with only one invert repeat region) alignment. The brown triangles represent the tandem repeat regions. (PDF 1013 kb)

Additional file 9: **Figure S6.** Aligned sequence matrix of *ycf2* gene shows the duplication of tandem repeat in *Holcoglossum*. (JPG 727 kb)

## Abbreviations

HDM: The Hengduan Mountains
IRA/ IRB: Inverted repeat regions A/B
LSC: Large single copy region
ML: Maximum likelihood
ORF: Open Reading Frame
SSC: Small single copy region
