# Supplementary material for: Evolution of plastid genomes of Holcoglossum (Orchidaceae) with recent radiation
Source: BMC Evol Biol. 2019 Feb 26;19:63. doi: 10.1186/s12862-019-1384-5 (PMC6390633; doi:10.1186/s12862-019-1384-5)
Supplement: Supplementary file 2 — Table S1. List of genes identified in the plastid genomes of Holcoglossum. (DOCX 26 kb) [file 12862_2019_1384_MOESM2_ESM.docx]

Table S1 List of genes identified in the plastid genomes of *Holcoglossum*

| Category of Genes | Group of gene | Name of gene |
| --- | --- | --- |
| Self-replication | Ribosomal RNA genes | *rrn*4.5×2, *rrn*5×2, *rrn*16×2, *rrn*23×2 |
|  | Transfer RNA genes | *trn*C-GCA, *trn*D-GUC, *trn*E-UUC, *trn*F-GAA, *trn*G-UCC, *trn*G-GCC^a^, *trn*K-UUU^a^, *trn*L-UAA^a^, *trn*L-UAG, *trn*M-CAU, *trn*P-UGG, *trn*Q-UUG, *trn*R-UCU, *trn*S-GCU, *trn*S-GGA, *trn*S-UGA, *trn*T-UGU, *trn*T-GGU, *trn*VUACa, *trn*Y-GUA, *trn*W-CCA, *trn*fM-CAU, *trn*A-UGC^a^ ×2, *trn*H-GUG×2, *trn*I-CAU×2, *trn*I-GAU^a^ ×2, *trn*L-CAA×2, *trn*N-GUU×2, *trn*R-ACG×2, *trn*V-GAC×2 |
|  | Ribosomal protein (small subunit) | *rps*2, *rps*3, *rps*4, *rps*7×2, *rps*8, *rps*11, *rps*12b ×2, *rps*14, *rps*15, *rps*16a, *rps*18, *rps*19×2 |
|  | Ribosomal protein (large subunit) | *rpl*2^a^ ×2, *rpl*14, *rpl*16^a^, *rpl*20, *rpl*22, *rpl*23×2, *rpl*32, *rpl*33, *rpl*36 |
|  | RNA polymerase | *rpo*A, *rpo*B, *rpo*C1^a^, *rpo*C2 |
|  | Translational initiation factor | *inf*A |
| Genes for photosynthesis | Subunits of photosystem I | *psa*A, *psa*B, *psa*C, *psa*I, *psa*J, *ycf*3^b^, *ycf*4 |
|  | Subunits of photosystem II | *psb*A, *psb*B, *psb*C, *psb*D, *psb*E, *psb*F, *psb*H, *psb*I, *psb*J, *psb*K, *psb*L, *psb*M,  *psb*N, *psb*T, *psb*Z |
|  | Subunits of cytochrome | *pet*A, *pet*B^a^, *pet*D^a^, *pet*G, *pet*L, *pet*N |
|  | Subunits of ATP synthase | *atp*A, *atp*B, *atp*E, *atp*Fa, *atp*H, *atp*I |
|  | Large subunit of Rubisco | *rbc*L |
|  | Subunits of NADH dehydrogenase^c^ | _ |
| Other genes | Maturase | *mat*K |
|  | Envelope membrane protein | *cem*A |
|  | Subunit of acetyl-CoA | *acc*D |
|  | c-type cytochrome Synthesis gene | *ccs*A |
|  | ATP-dependent protease | *clp*P^b^ |
|  | Component of TIC complex | *ycf*1×2 |
|  | Genes of unknown function Conserved open reading frames | *ycf*2×2 |

×2—two gene copies in IR regions

a With one intron

b With two introns

c Detailed as following continued table

* continued table

|  | *ndh*A | *ndh*B | *ndh*C | *ndh*D | *ndh*E | *ndh*F | *ndh*G | *ndh*H | *ndh*I | *ndh*J | *ndh*K |
| --- | --- | --- | --- | --- | --- | --- | --- | --- | --- | --- | --- |
| *Holcoglossum nujiangense*_S1_S16 | - | ψ(2061) | - | ψ(1154) | ψ(306) | - | ψ(535) | - | - | - | - |
| *Holcoglossum nujiangense*_S5_S9 | - | ψ(2061) | - | ψ(1144) | ψ(306) | - | ψ(535) | - | - | - | - |
| *Holcoglossum weixiense* | - | ψ(2024) | - | ψ(1223) | ψ(306) | - | ψ(536) | - | - | - | - |
| *Holcoglossum sinicum* | - | ψ(2054) | - | ψ(905) | ψ(306) | - | ψ(538) | - | - | - | - |
| *Holcoglossum flavescens* _S2_S18 | - | ψ(2056) | - | ψ(1207) | ψ(306) | - | ψ(535) | - | - | - | - |
| *Holcoglossum flavescens*_S5_S10 | - | ψ(2056) | - | ψ(1207) | ψ(306) | - | ψ(535) | - | - | - | - |
| *Holcoglossum rupestre* | - | ψ(2054) | - | ψ(1231) | ψ(306) | - | ψ(535) | - | - | - | - |
| *Holcoglossum quasipinifolium* | - | ψ(2056) | ψ(354) | ψ(1119) | ψ(306) | - | ψ(534) | - | - | ψ(399) | ψ(472) |
| *Holcoglossum lingulatum* | - | ψ(2037) | ψ(354) | ψ(1121) | ψ(306) | - | - | - | - | ψ(436) | ψ(472) |
| *Penkimia nagalandensis* | - | ψ(2043) | - | ψ(1144) | ψ(320) | - | ψ(201) | - | - | - | - |
| *Holcoglossum amesianum* | - | ψ(2044) | - | ψ(1164) | ψ(297) | - | ψ(535) | - | - | - | - |
| *Ascocentrum himalaicum* | - | ψ(1558) | - | ψ(1153) | ψ(125) | - | ψ(535) | - | - | - | - |
| *Holcoglossum wangii* | - | ψ(2052) | - | ψ(1144) | ψ(124) | - | ψ(536) | - | - | - | - |
| *Holcoglossum subulifolium* | - | ψ(2031) | - | ψ(1151) | ψ(306) | - | ψ(534) | - | - | - | - |
| *Neofinetia falcata* | - | ψ(2059) | - | ψ(987) | ψ(305) | - | ψ(536) | - | - | - | - |
| *Neofinetia richardsiana* | - | ψ(2059) | - | ψ(987) | ψ(305) | - | ψ(536) | - | - | - | - |
| *Vanda brunnea* | - | ψ(2021) | ψ(371) | ψ(1002) | ψ(306) | - | ψ(536) | - | - | ψ(460) | ψ(489) |

In this table, “-” and “ψ” orderly represent gene loss and pseudogenization. The number in parentheses represents the length of identified *ndh*-gene homologous block.
