# Supplementary figures and images for: Evolution of plastid genomes of Holcoglossum (Orchidaceae) with recent radiation
Source: BMC Evol Biol. 2019 Feb 26;19:63. doi: 10.1186/s12862-019-1384-5 (PMC6390633; doi:10.1186/s12862-019-1384-5)

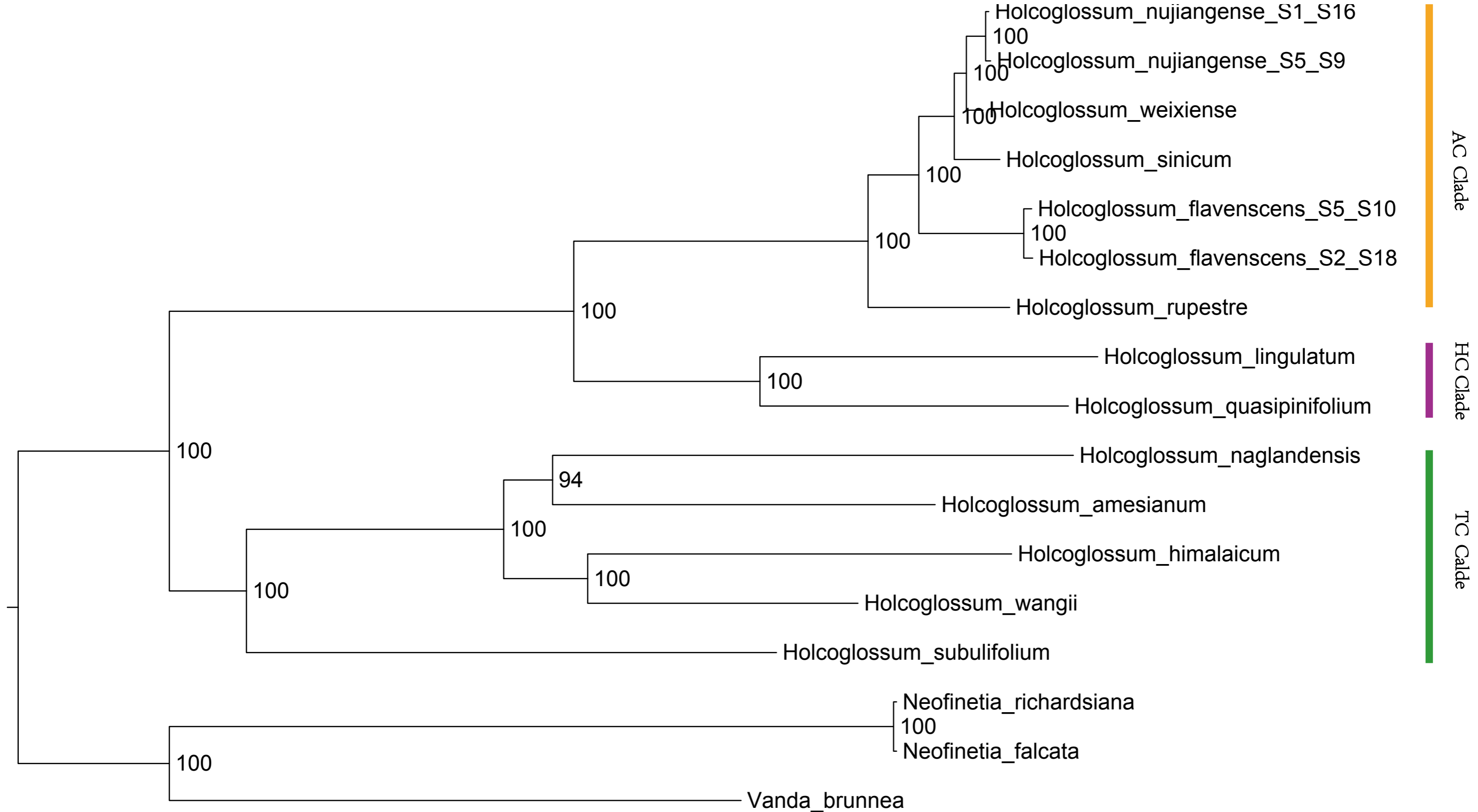

0.002

Supplement: Supplementary file 3 — Figure S1. Maximum Likelihood phylogenetic tree of Holcoglossum based on the whole plastid genome except for one invert repeat region. Bootstrap support is indicated on the nodes. (PDF 171 kb) [file 12862_2019_1384_MOESM3_ESM.pdf]

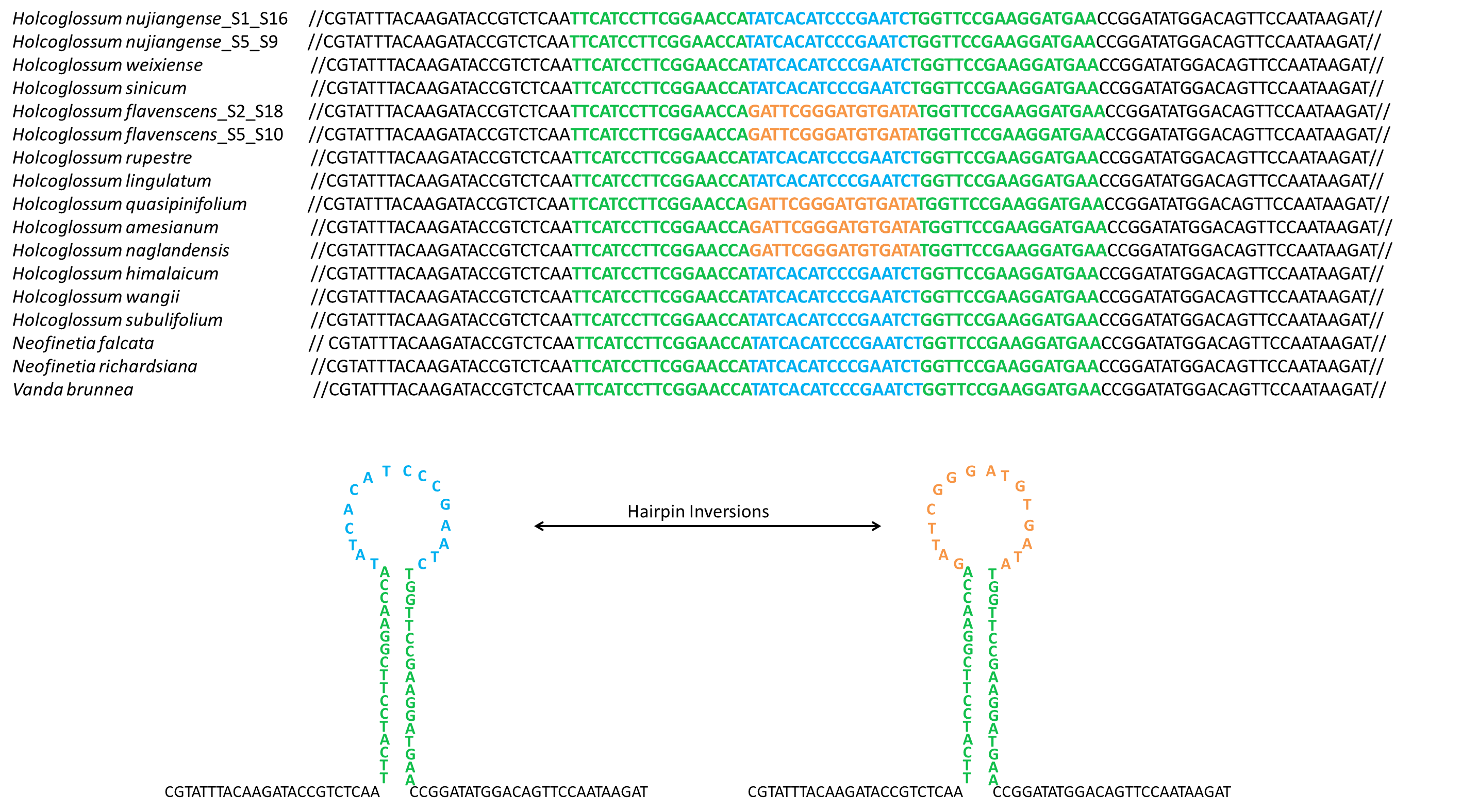

Supplement: Supplementary file 5 — Figure S2. Hairpin inversion of ycf2 in Holcoglossum. (PNG 236 kb) [file 12862_2019_1384_MOESM5_ESM.png]

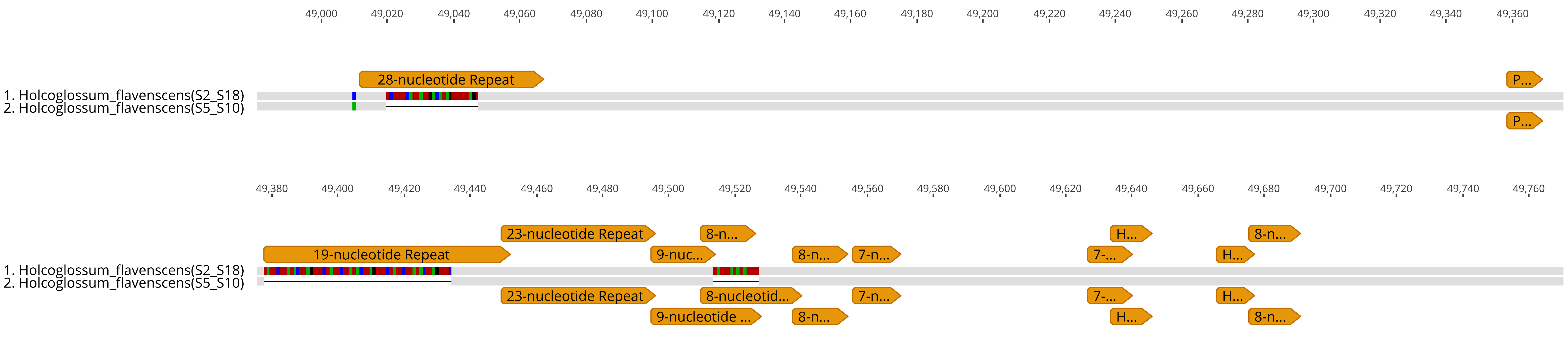

Supplement: Supplementary file 7 — Figure S4. Intraspecific variation resulting from tandem repeats in H. flavenscens. (PNG 201 kb) [file 12862_2019_1384_MOESM7_ESM.png]

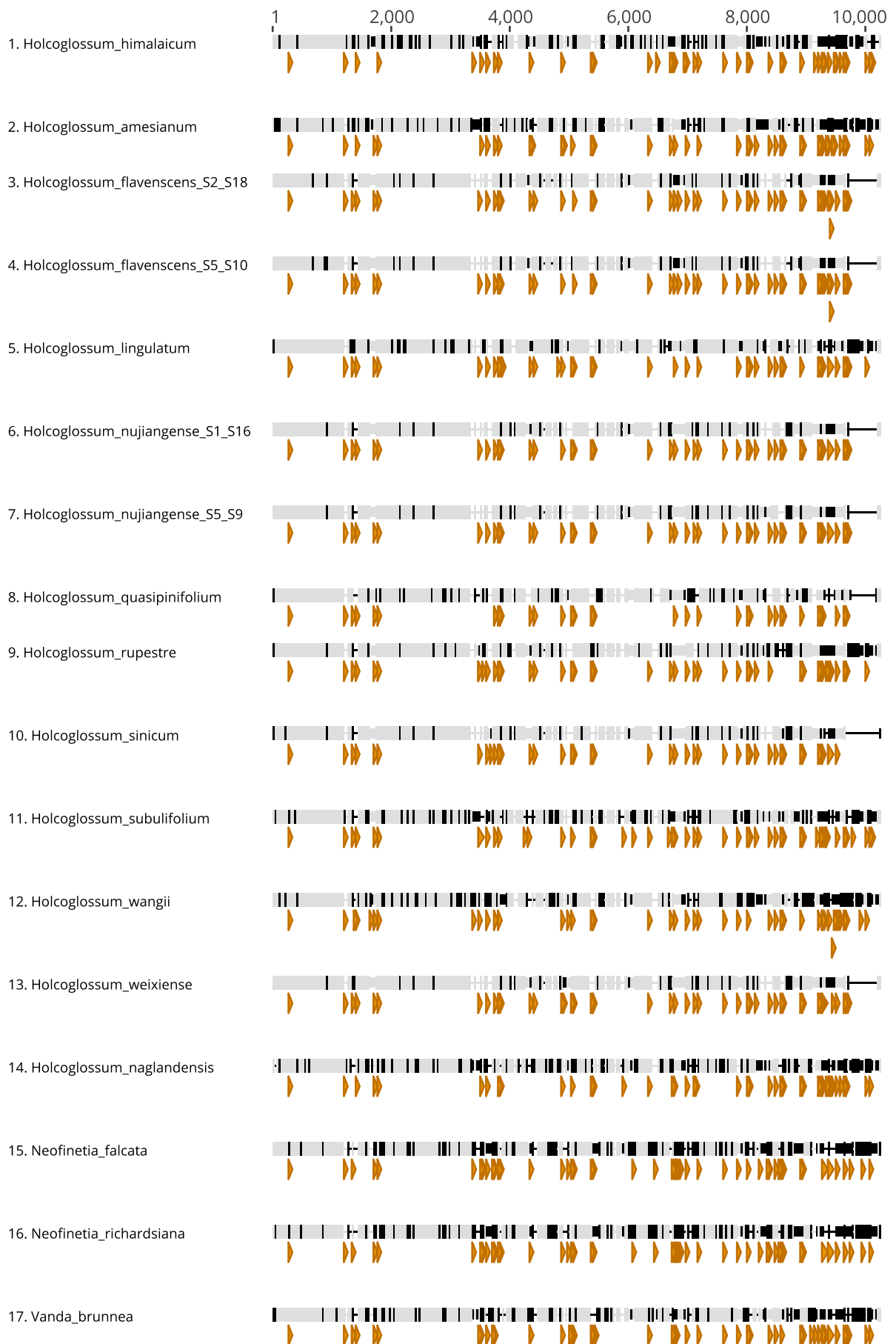

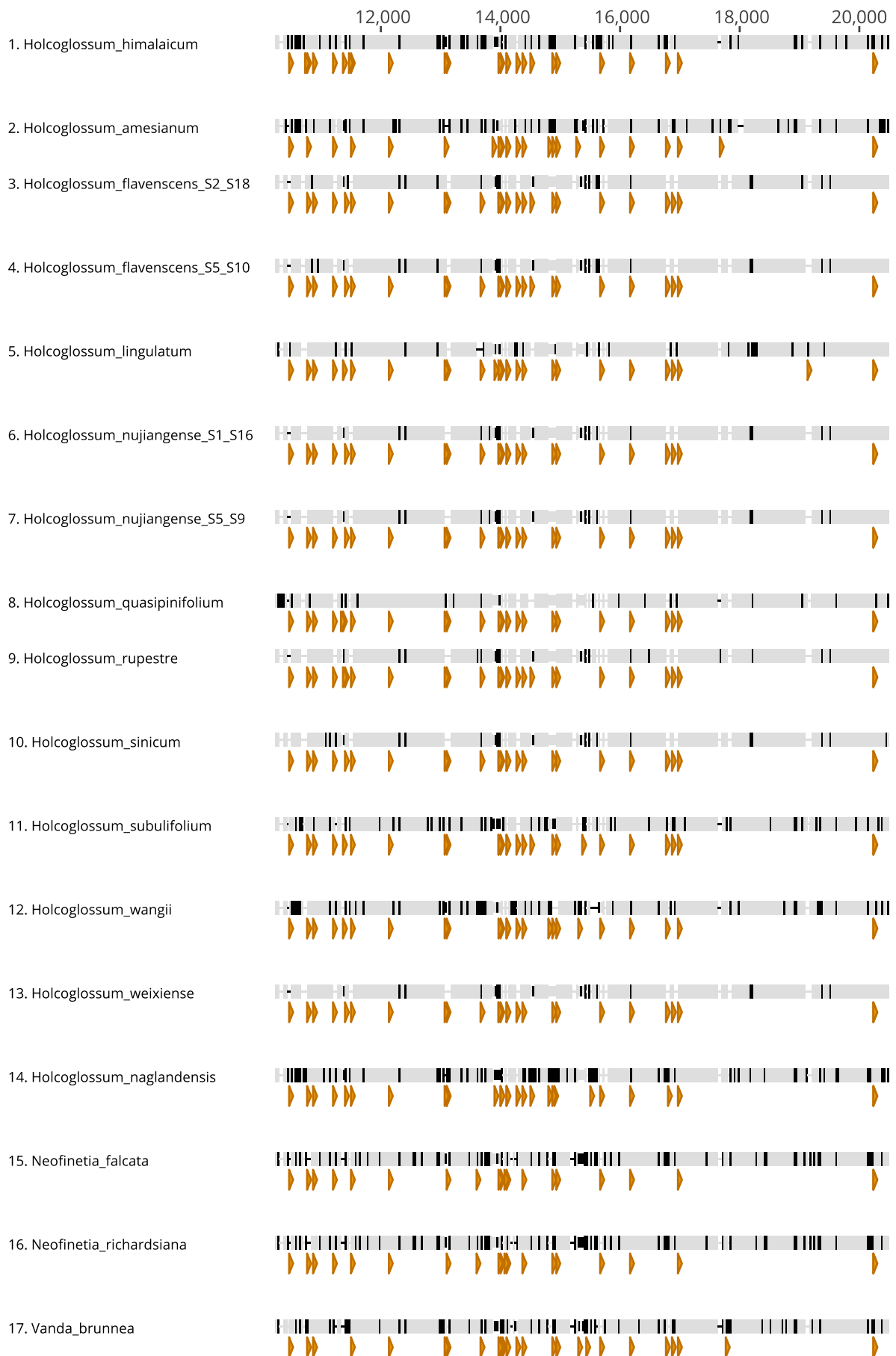

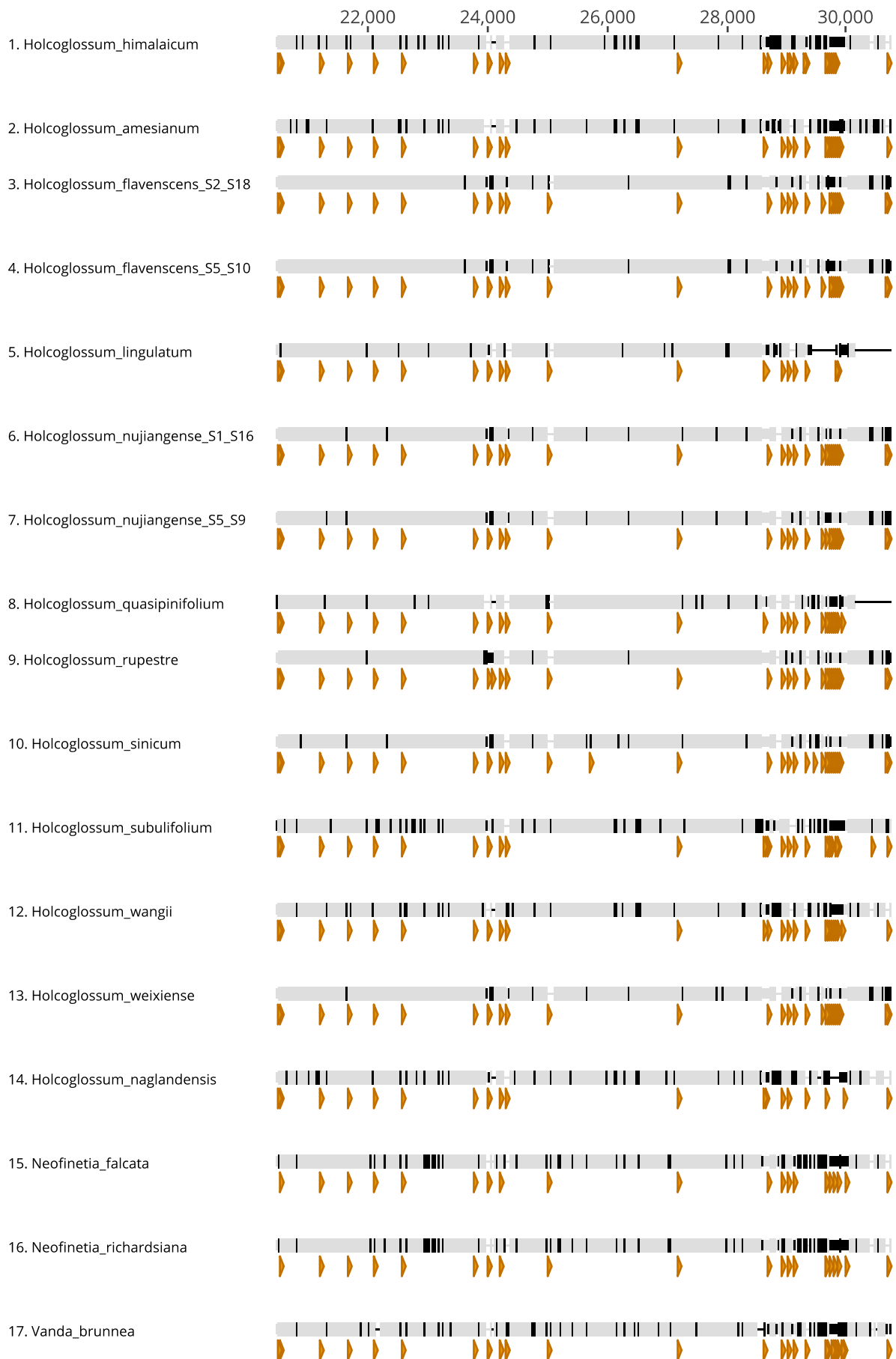

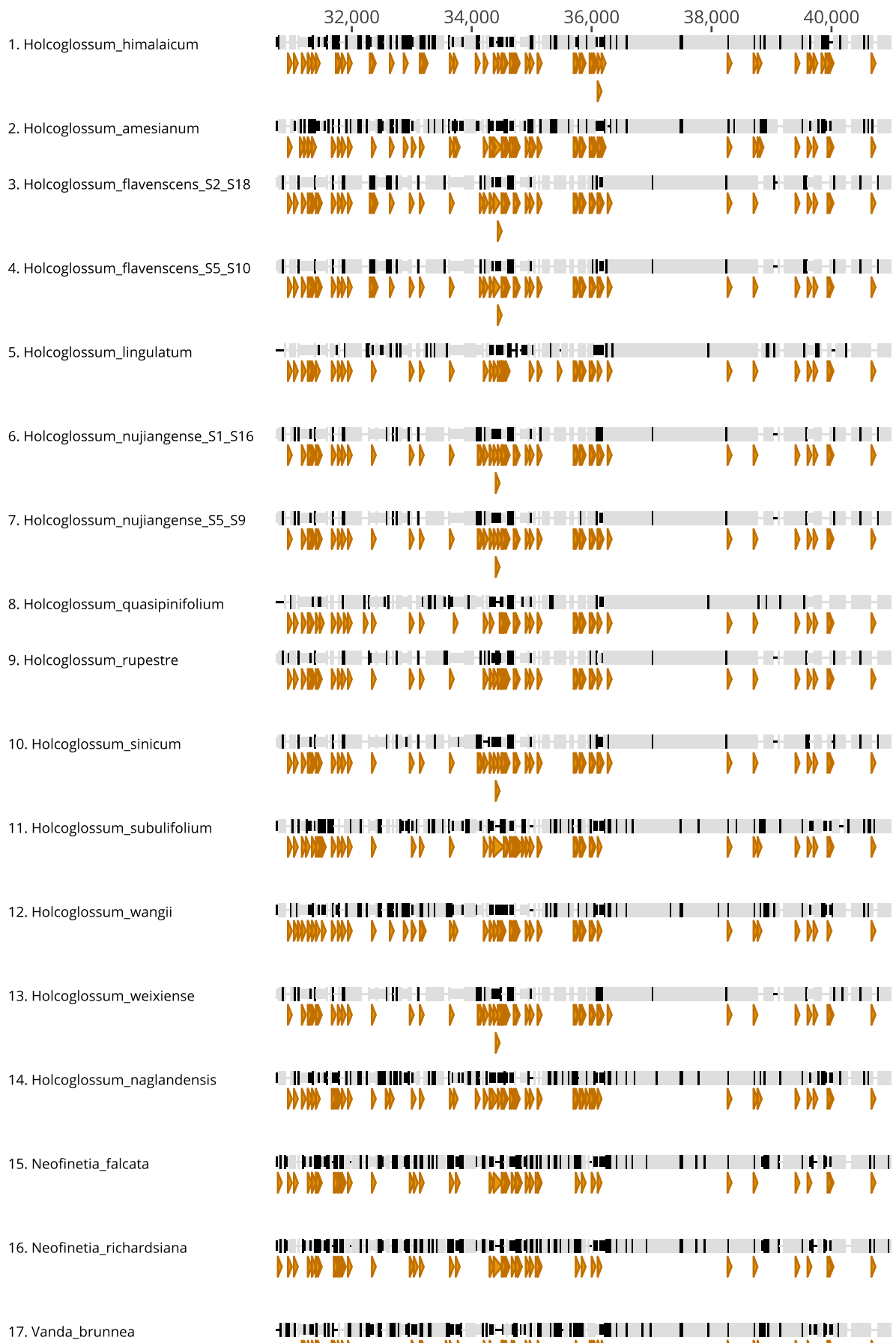

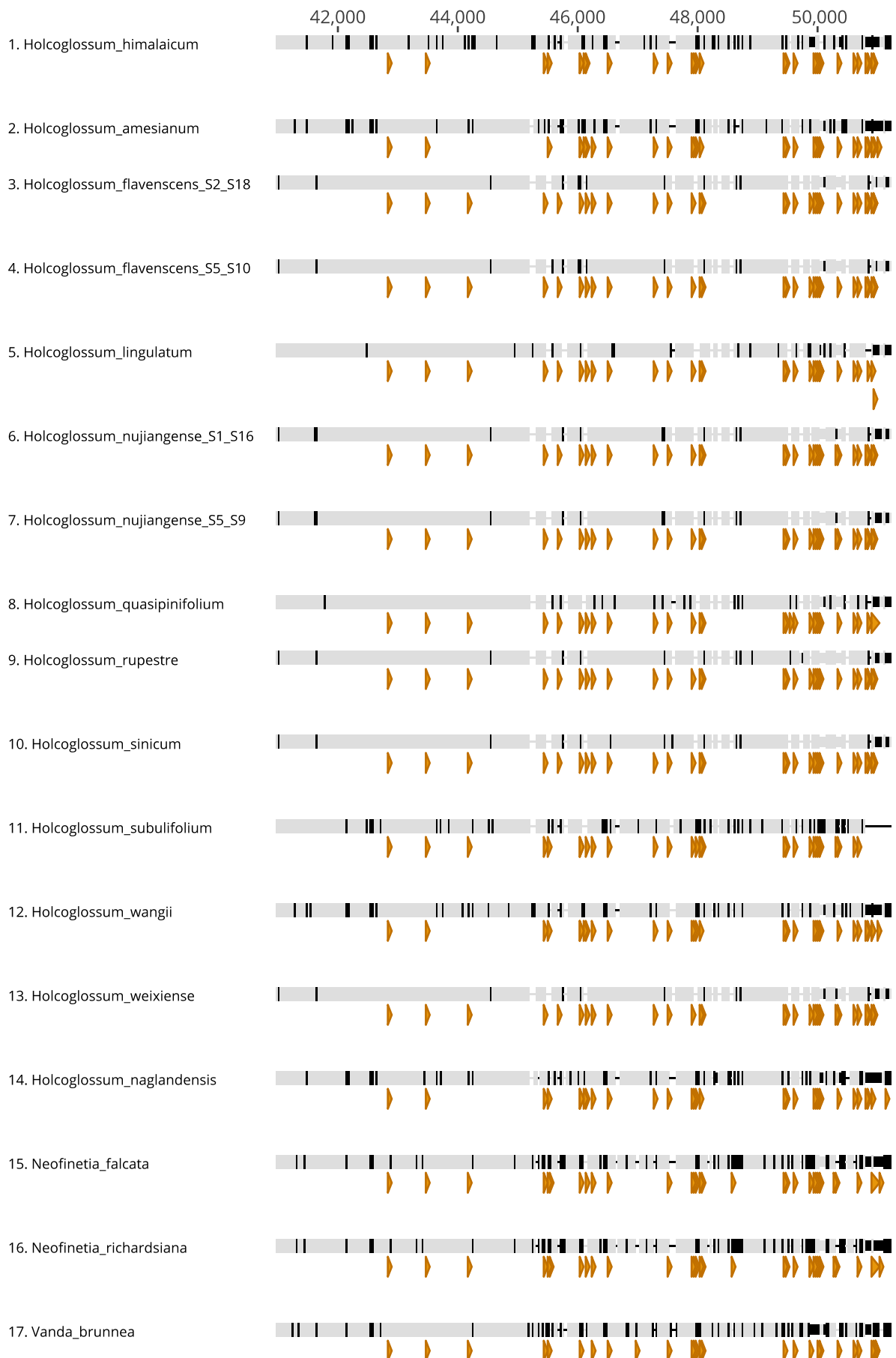

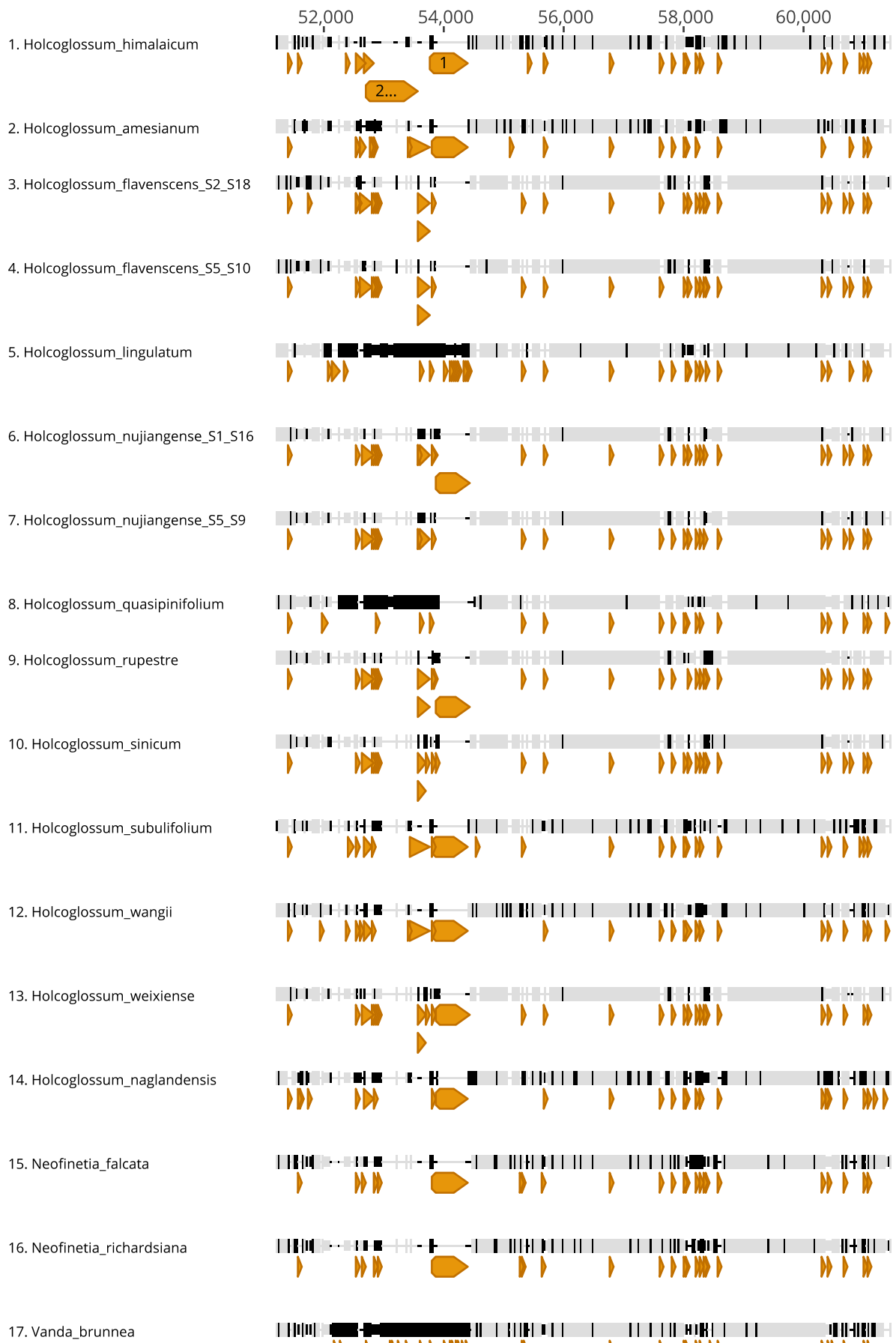

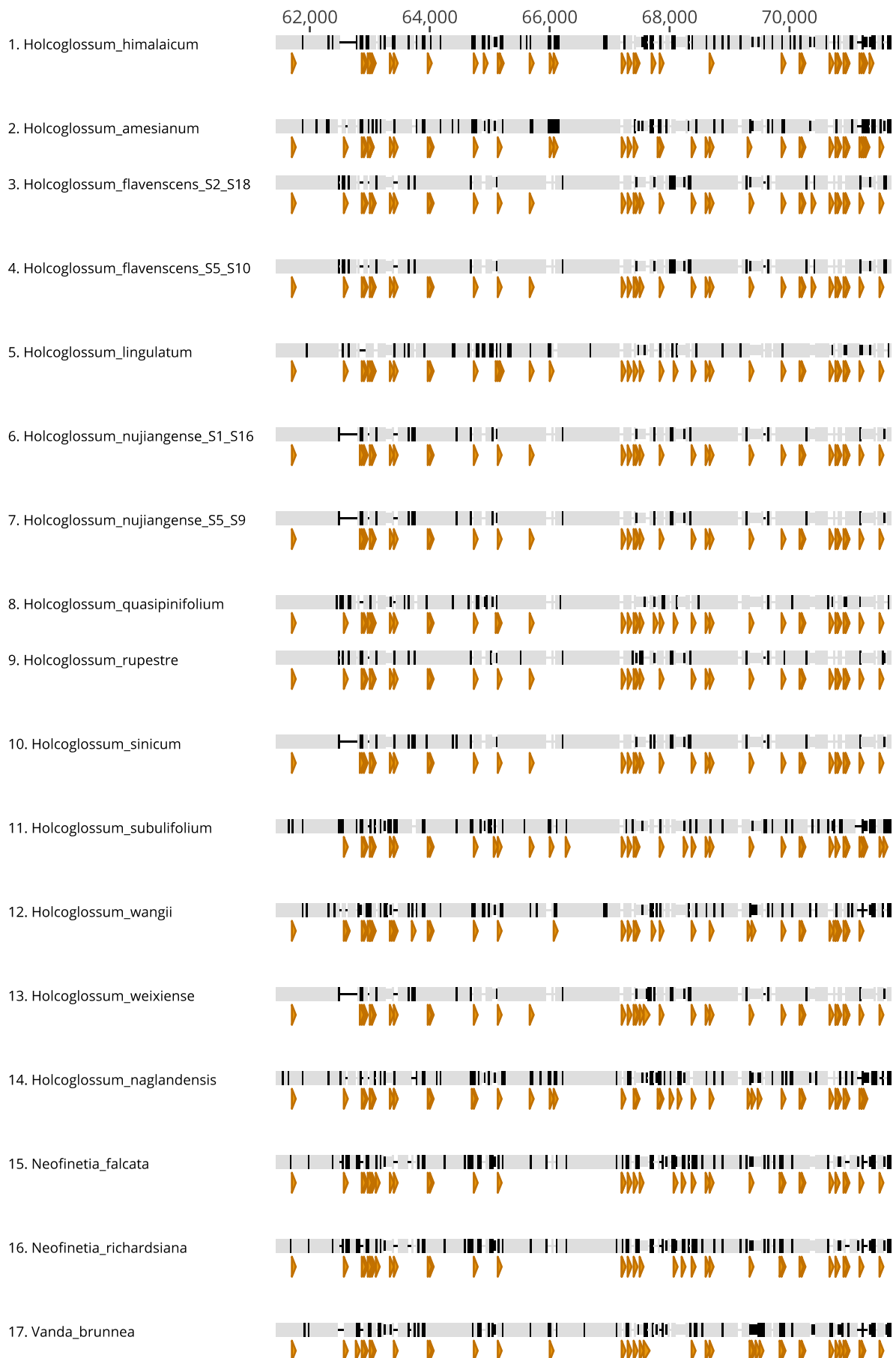

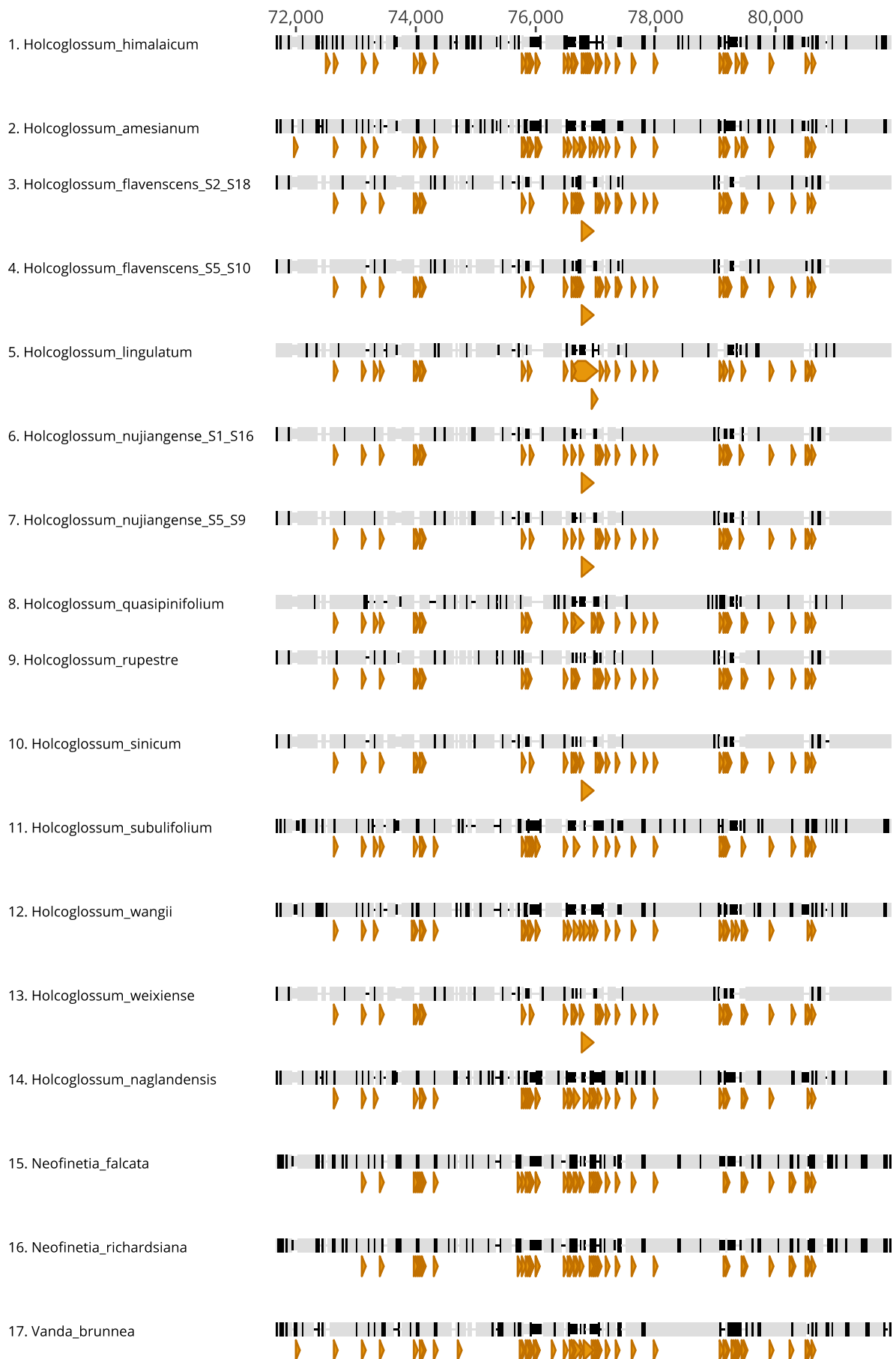

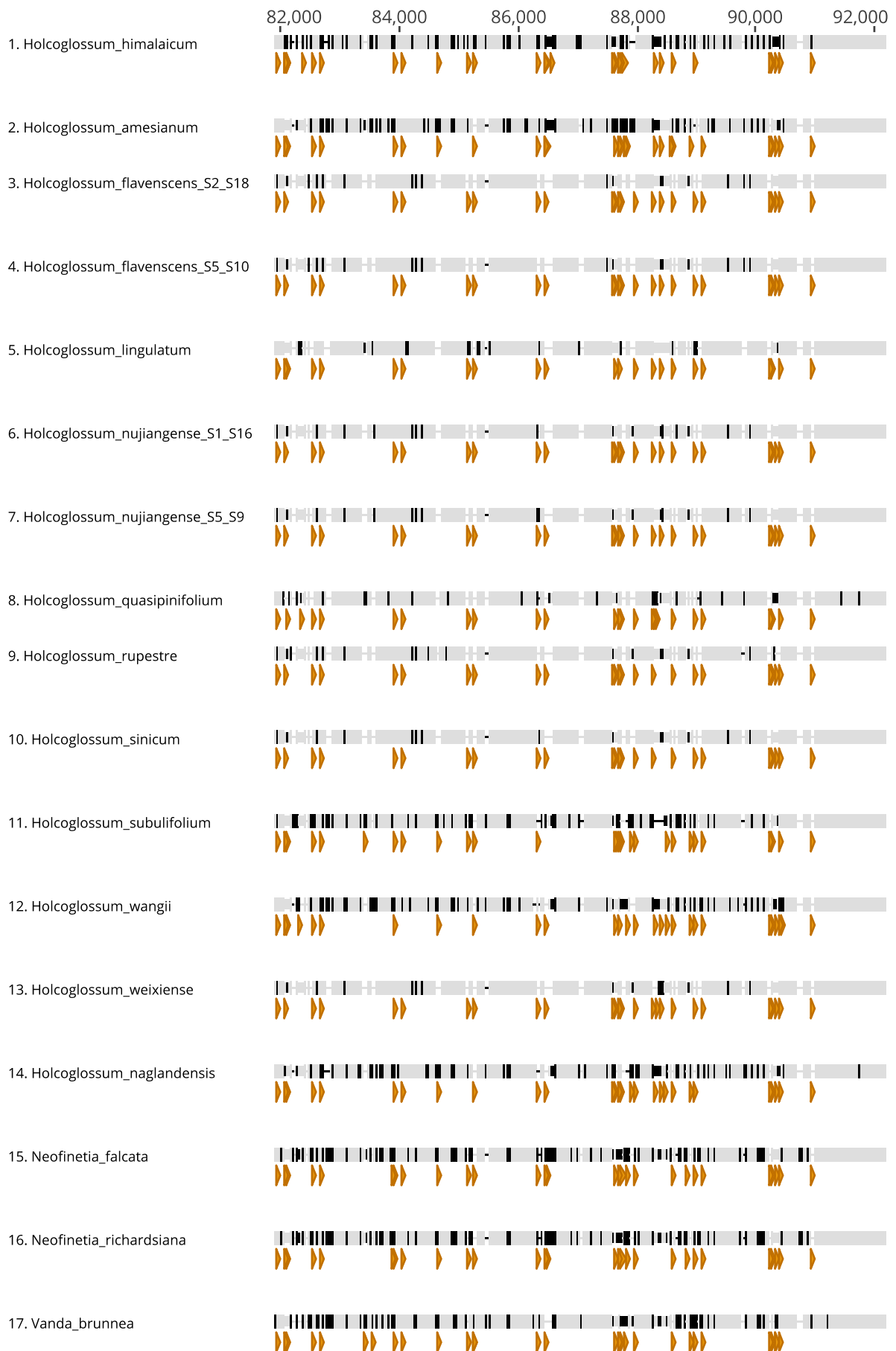

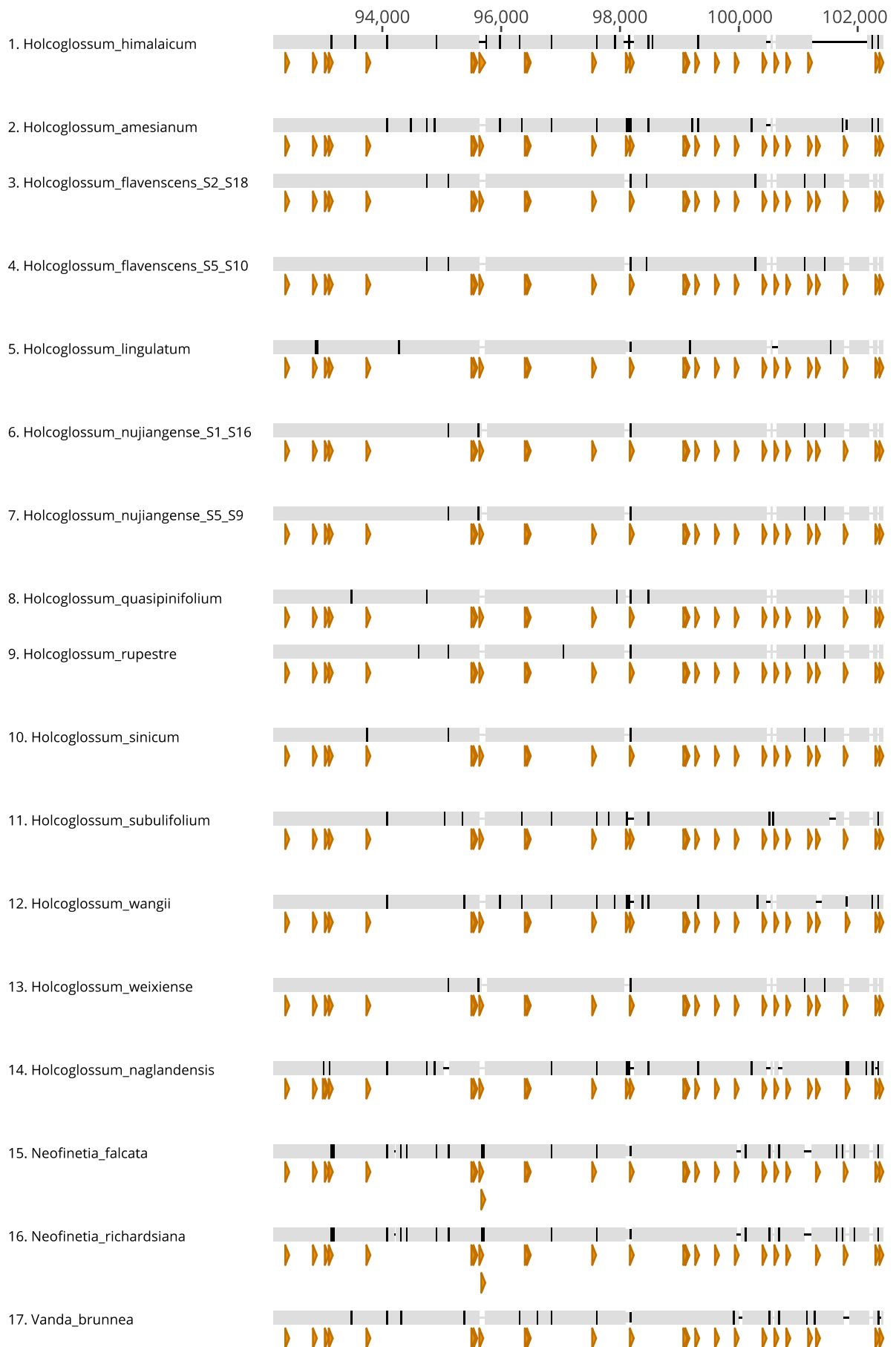

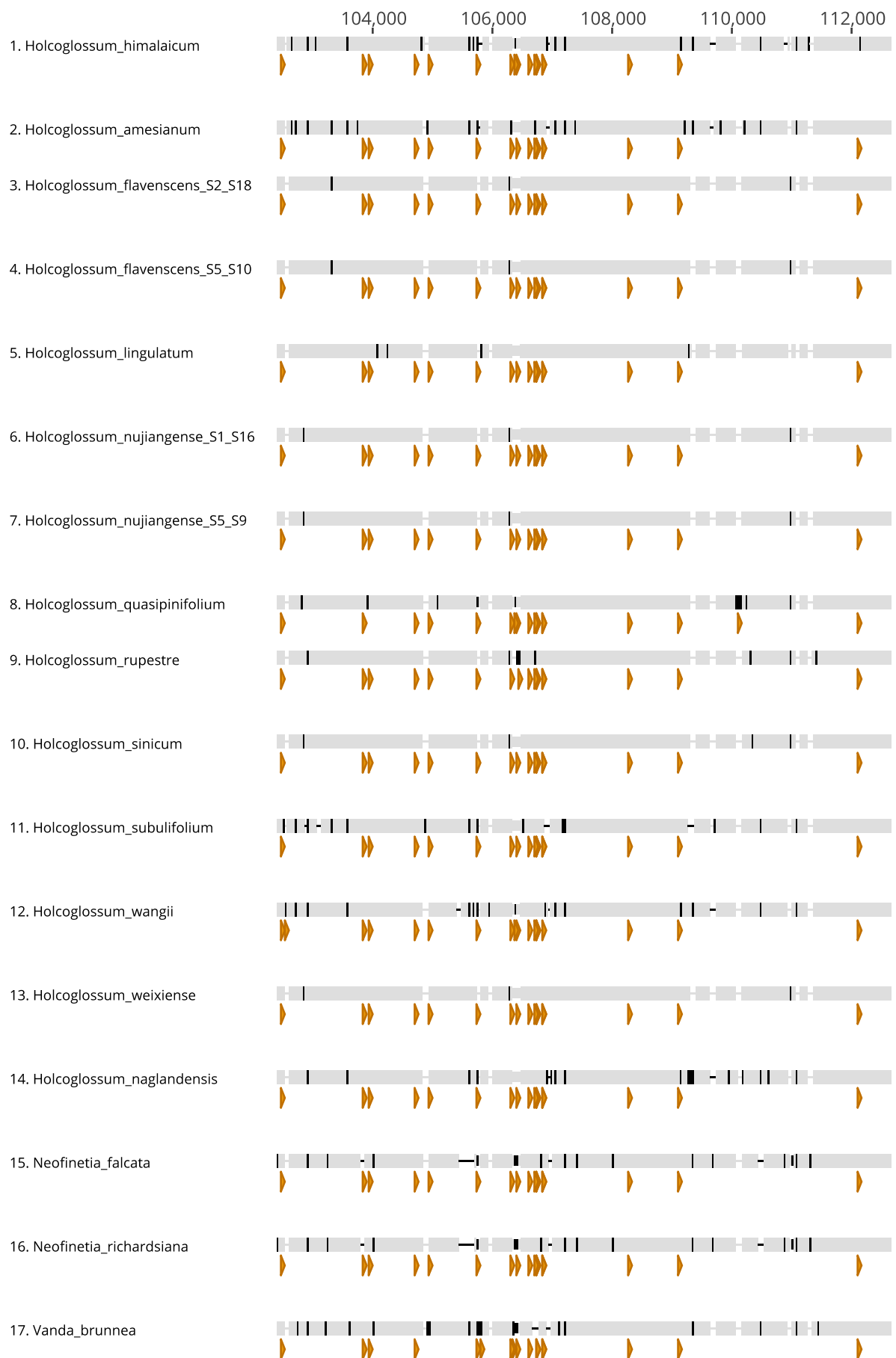

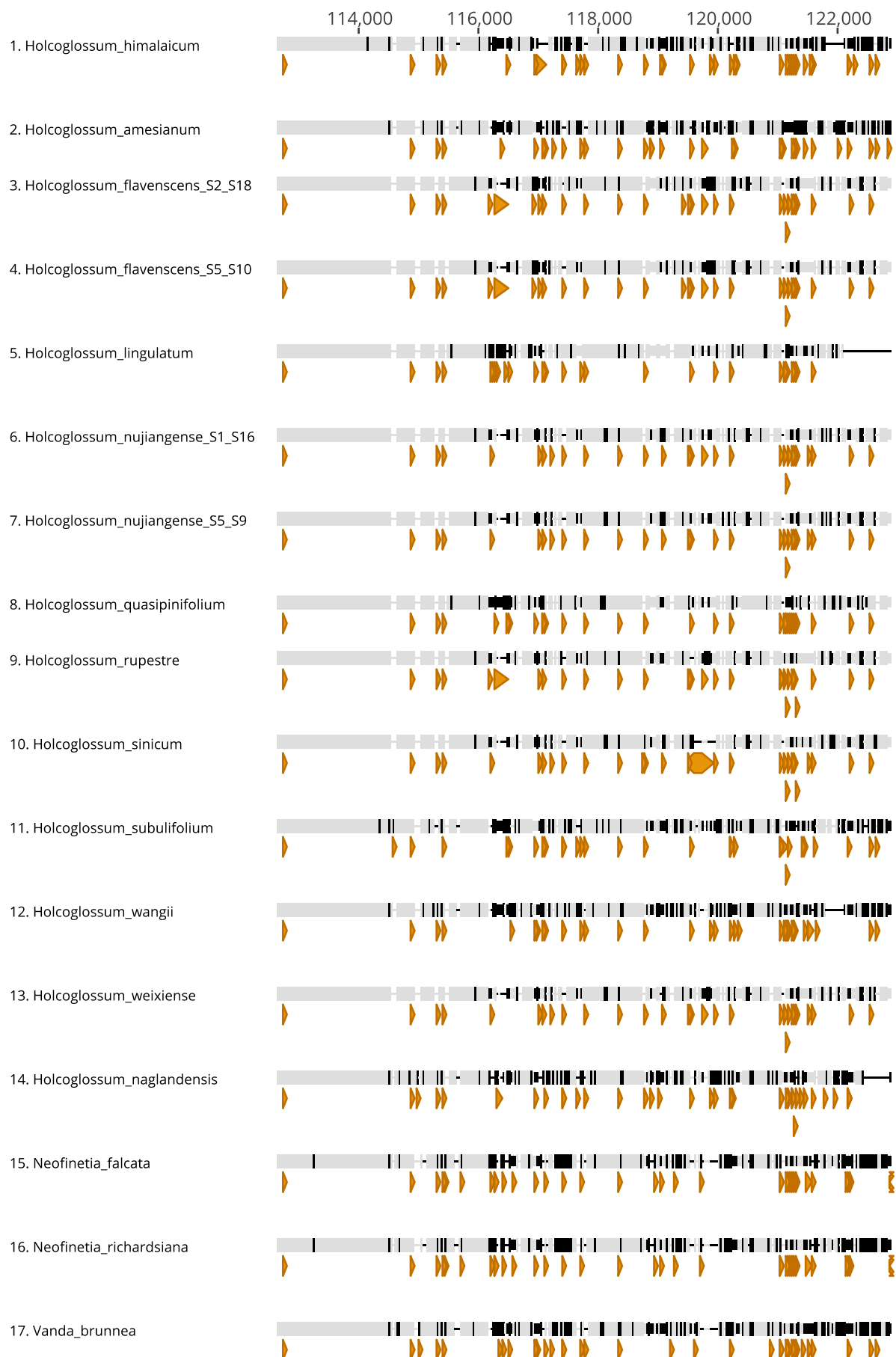

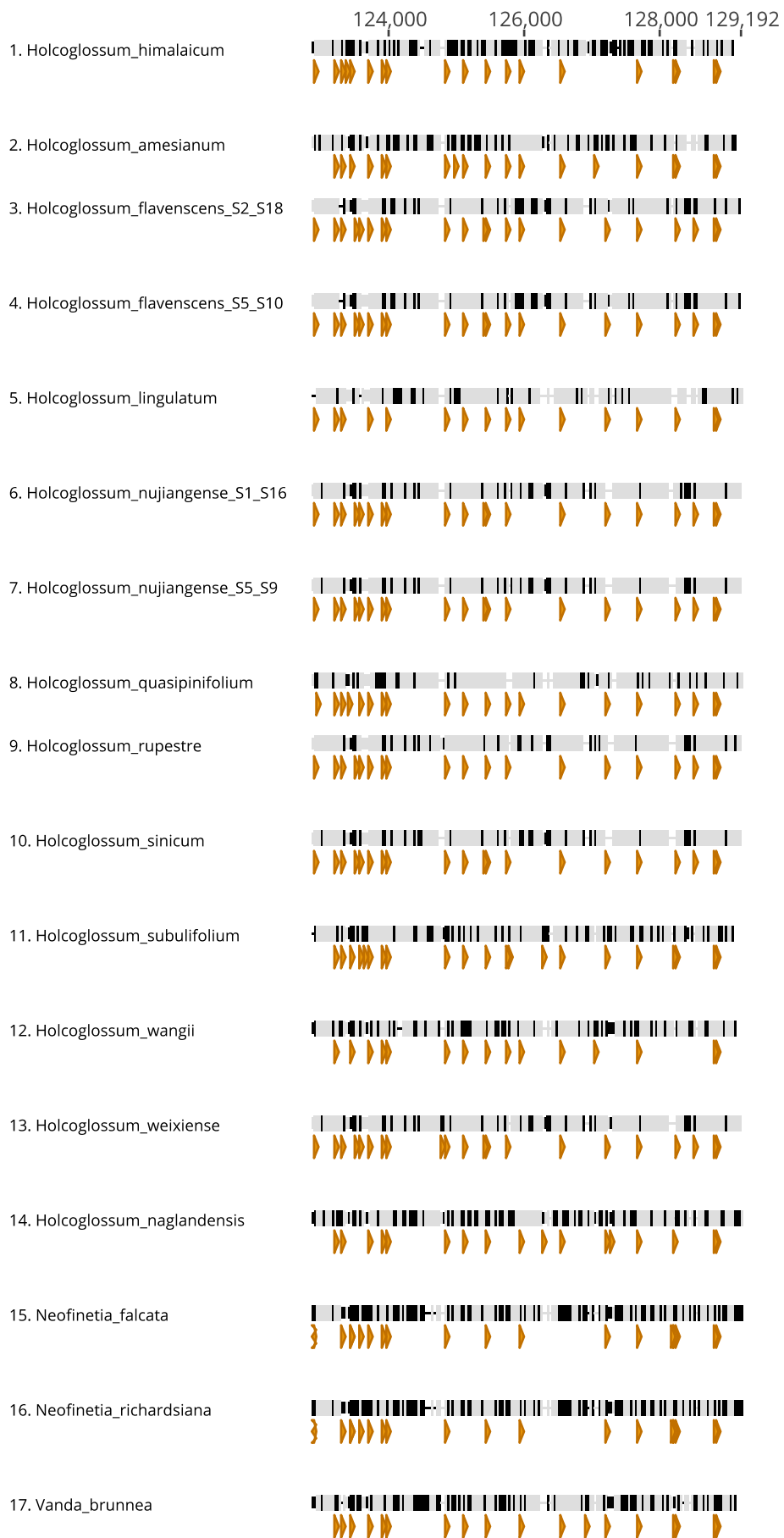

Supplement: Supplementary file 8 — Figure S5. Tandem repeat annotated to the whole plastid genome (with only one invert repeat region) alignment. The brown triangles represent the tandem repeat regions. (PDF 1013 kb) [file 12862_2019_1384_MOESM8_ESM.pdf]

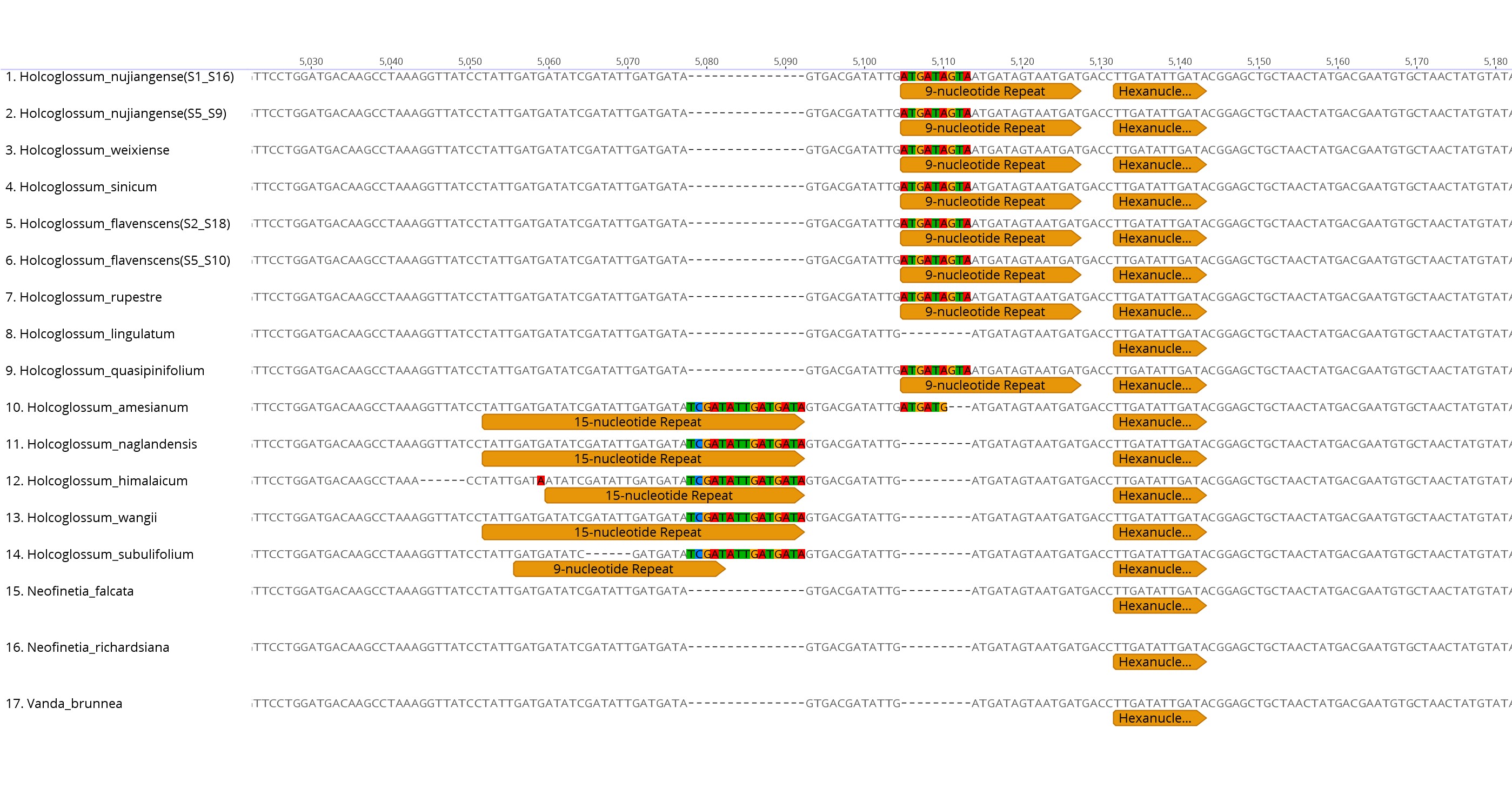

Supplement: Supplementary file 9 — Figure S6. Aligned sequence matrix of ycf2 gene shows the duplication of tandem repeat in Holcoglossum. (JPG 727 kb) [file 12862_2019_1384_MOESM9_ESM.jpg]
