# Supplementary material for: Evolution of plastid genomes of Holcoglossum (Orchidaceae) with recent radiation
Source: BMC Evol Biol. 2019 Feb 26;19:63. doi: 10.1186/s12862-019-1384-5 (PMC6390633; doi:10.1186/s12862-019-1384-5)
Supplement: Supplementary file 4 — Table S3. Detected positive selection sites in the plastid genes of TC clade Holcoglossum species. (DOCX 19 kb) [file 12862_2019_1384_MOESM4_ESM.docx]

**Table S3.** Detected positive selection sites in the plastid genes of TC clade *Holcoglossum* species

| **Gene** | **Branch-site model** | **-lnL** | **ω Values** | | | ***P* Value** | **Positively selected sites** |
| --- | --- | --- | --- | --- | --- | --- | --- |
|  |  |  |  |  |  |  | Site: BEB value |
| *ycf1* | Alternative | -9518.383 | ω0=0 | ω1=1 | ω2=10.01 | <0.005 | 786 0.976* |
|  | Null | -9530.054 | ω0=0.031 | ω1=1 | ω2=1 |  | 1486 0.992** |
|  |  |  |  |  |  |  | 1524 0.937 |
|  |  |  |  |  |  |  |  |
| *ycf2* | Alternative | -10053.0 | ω0=0 | ω1=1 | ω2=174.32 | <0.005 | 560 0.966* |
|  | Null | -10137.676 | ω0=0.031 | ω1=1 | ω2=1 |  | 561 0.926 |
|  |  |  |  |  |  |  | 562 0.949 |
|  |  |  |  |  |  |  | 662 0.993** |
|  |  |  |  |  |  |  | 663 0.996** |
|  |  |  |  |  |  |  | 664 0.961* |
|  |  |  |  |  |  |  | 948 0.966* |
|  |  |  |  |  |  |  | 1063 0.996** |
|  |  |  |  |  |  |  | 1652 0.966* |
|  |  |  |  |  |  |  | 1653 0.963* |
|  |  |  |  |  |  |  | 1654 0.965* |
|  |  |  |  |  |  |  | 1656 0.964* |
|  |  |  |  |  |  |  | 1660 1.000** |
|  |  |  |  |  |  |  | 1661 1.000** |
|  |  |  |  |  |  |  | 1665 1.000** |
|  |  |  |  |  |  |  | 1666 1.000** |

*P*-value is the results of LRT test. Sites identified as positively selected by Bayes empirical Bayes (BEB) analysis with posterior probability (PP) >= 0.90 are listed.
